# Conserved role of primary motor cortex in the control of prehension in mice and macaques

**DOI:** 10.1016/j.celrep.2026.117419

**Published:** 2026-06-01

**Authors:** Francisco Aparicio, Harman Ghuman, Preeya Khanna, Sapeeda Barati, Robert Morecraft, Christoph Kirst, Karunesh Ganguly

**Affiliations:** 1 Department of Neurology, University of California, San Francisco, San Francisco, CA 94158, USA; 2 San Francisco VA Medical Center, San Francisco, CA 94158, USA; 3 Department of Anatomy, University of California, San Francisco, San Francisco, CA 94158, USA; 4 California National Primate Research Center, University of California, Davis, Davis, CA 95616, USA; 5 Laboratory of Neurological Sciences, Division of Biomedical and Translational Sciences, Sanford School of Medicine, The University of South Dakota, Vermillion, SD 57069, USA; 6 These authors contributed equally; 7 Lead contact

## Abstract

A central goal in neuroscience is to develop cross-species frameworks that reveal conserved principles of movement control. Recent work has suggested a potential species difference in the role of primary motor cortex (M1) during skilled forelimb tasks: in rodents, extensive practice appears to shift control to subcortical circuits, leading to “disengagement” of M1 from task execution. Importantly, there is no evidence of such disengagement in macaques. We hypothesized that these differences instead reflect task demands, particularly the need for fine (i.e., grasping) versus gross motor control. Strikingly, M1 lesions in macaques produced recovery patterns similar to rodents: gross motor control, including an exclusively gross motor task, recovered rapidly, whereas fine motor control exhibited persistent deficits. Detailed kinematic analyses in both macaques and mice performing a single-pellet reach-to-grasp task (RGT) revealed analogous disruptions in the temporal structure of sub-movements. These findings underscore the importance of task design and the translational value of rodent models for understanding motor control and recovery.

## In brief

Aparicio and Ghuman et al. show that primary motor cortex (M1) lesions cause rapid recovery of reaching but prolonged grasping deficits in both mice and macaques. These conserved behavioral effects are accompanied by stable engagement of deep-layer M1 projection neurons during long-term skilled motor training.

## Graphical Abstract

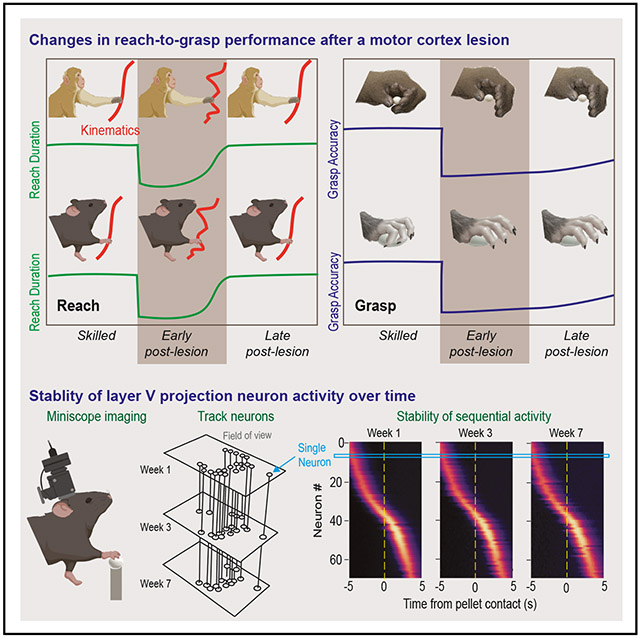

## INTRODUCTION

A key objective in neuroscience is to develop comparative frameworks across species to deepen our understanding of principles of motor control and the potential for recovery after injury.^1-4^ When considering classic literature and more recent studies,^5-9^ there appears to be a fundamental difference between rodents and macaques regarding the essential role of primary motor cortex (M1) in the execution of well-practiced skills using the forelimb. Here, we test the hypothesis that these apparent species-specific differences may instead reflect differences in task demand, particularly the requirement for fine (e.g., prehension) versus gross (e.g., reaching) motor control.

Recent studies in both rats and mice suggest that with extended practice of gross motor tasks, movement control shifts from M1 to subcortical structures,^7-10^ resulting in functional “disengagement” of M1. Disengagement refers to the relative absence of a performance deficit following either a permanent M1 lesion^7^ (but allowing time for compensatory plasticity^11^) or acute optogenetic inhibition.^8^ Additional evidence for disengagement comes from chronic recordings showing reduced task-related activity in cortical layers 2/3 of M1 following long-term training.^8^ However, this phenomenon of disengagement is not universally observed for all forelimb tasks.^12^ Studies have also demonstrated that both cortical and deep-layer-specific optogenetic inactivation of M1 markedly impairs reach-to-grasp performance, i.e., require a combination of gross and fine motor control.^13,14^ Similarly, a substantial body of literature has found that M1 lesions in rodents can produce prolonged deficits in reach-to-grasp tasks (RGTs).^15-19^ However, most of these studies did not explicitly control for the extent of pre-lesion training and practice. Thus, it remains unclear if, in rodents, all forelimb tasks will demonstrate functional disengagement after extensive practice.

In contrast, there is little evidence of disengagement in primates. For non-human primates (NHPs) and humans, lesions of the motor cortex result in prolonged deficits of gross and fine motor control.^4-6,20-24^ Notably, classic lesion studies in NHPs found that extensive motor cortical lesions led to prolonged deficits of fine motor control^5^; while there was relative recovery of gross motor control, the time course is unclear and it remains unknown if recovered kinematics matched those prior to the lesion. Many studies have also found that M1 lesions result in deficits of the RGT control.^6,21,22,25-27^ Together, this indicates that in primates, tasks involving both gross and fine motor control do not appear to show evidence of disengagement. Because it remains unclear whether an exclusively gross motor task might parallel the findings in rodents, the question of whether there are genuinely species-specific differences remains unclear.

We thus specifically tested the hypothesis that differences in the requirement for fine versus gross forelimb control contribute to apparent species-specific differences. To test this, we first examined the effects of M1 lesions in macaques on largely isolated gross and fine motor control tasks; this data was collected as a part of a study testing brain stimulation methods after brain lesions.^25^ Strikingly, gross motor control was initially affected but then recovered relatively rapidly and in a window that mirrored the findings in rats.^7^ In contrast, there were prolonged fine motor control deficits. We then conducted a comparative study in macaques and mice using the single-pellet RGT. M1 lesions resulted in surprising similarities: prehension deficits persisted longer than reaching deficits, and both species exhibited similar disruptions in the reliability of reach-to-grasp transitions. These results suggest that M1 plays a relatively conserved role in motor control in both mice and macaques. We also hypothesized that M1 projection neurons, which are known to be important for execution, are more likely to show persistent task-related engagement. We found that layer 5 projection neurons maintained stable activation over extended periods of training, further supporting the notion of long-term M1 engagement in the reach/grasp task. More broadly, our study emphasizes the necessity of accounting for the differences between fine and gross motor control in efforts to elucidate fundamental motor control principles across species.

## RESULTS

### Transient deficits in a reaching task in NHPs

**Figure 1.**
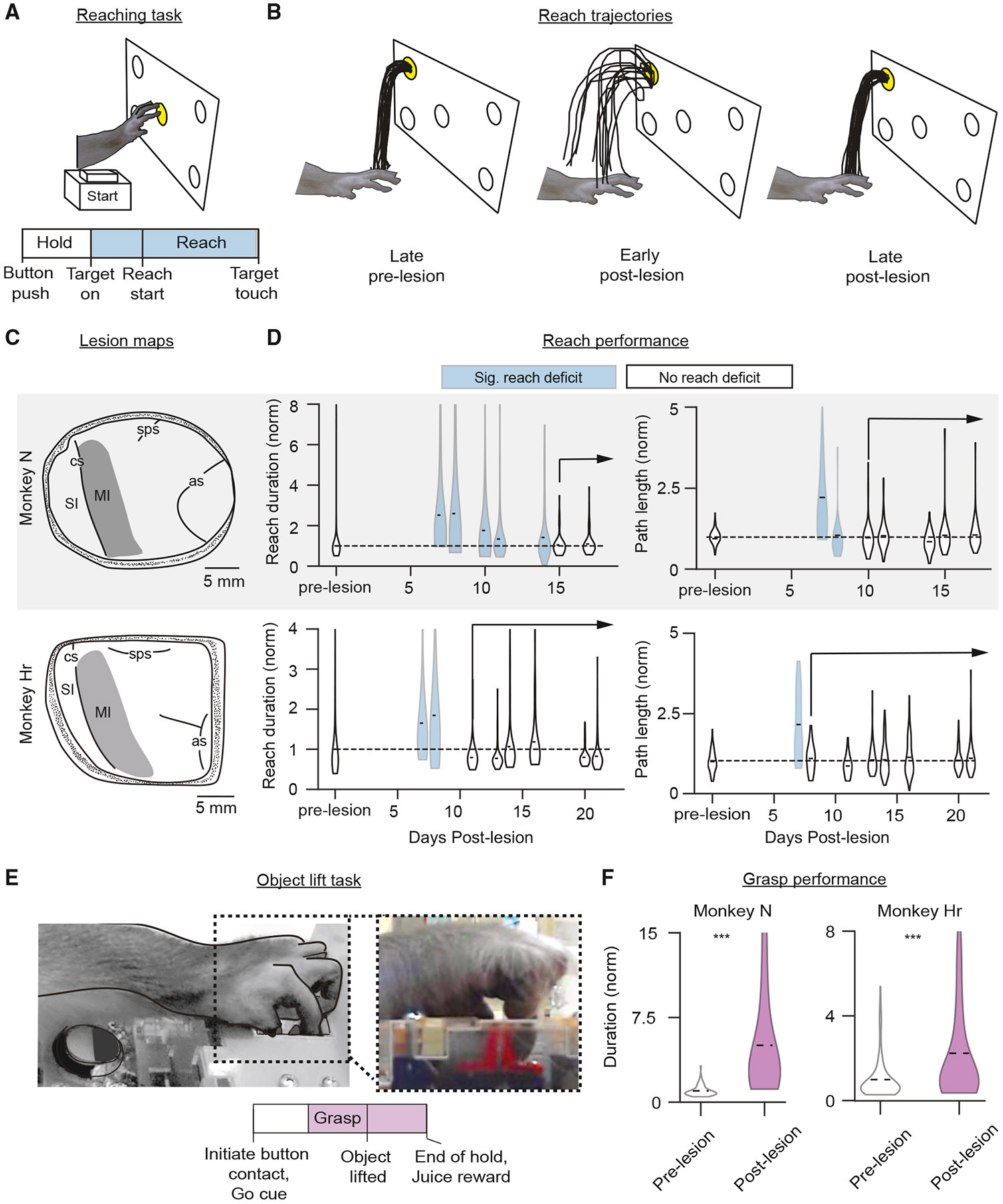
Recovery of reach performance after short-term reach deficits in NHPs In the target-touch task experimental setup, the animals were trained on a dedicated touchscreen reaching task where they were tasked with touching one of the five targets on a screen. The monkeys initiate the trial by pressing the button (to make the target appear), and the trial ended after they touched the target. The experiment consisted of a pre-lesion learning stage and a post-lesion recovery stage. The animals went 7 days without behavior testing following the lesion (days 0–6). We measured the reach duration (from reach onset to target touch) and the path lengths (the length of their trajectory to the target). (A) Schematic of the experimental design for the touchscreen reaching task. (B) Example reach trajectories pre- (left) and early post-lesion (middle) and late post-lesion (right). (C) Lesion maps for monkey N (top) and monkey Hr (bottom). as, arcuate sulcus; cs, central sulcus; M1, primary motor cortex; S1, primary somatosensory cortex; sps, superior precentral sulcus. Scale bars, 5mm. (D) Violin plots illustrating the distribution of reach durations and path lengths (pre- and post-lesion) for monkey N (top) and monkey Hr (bottom). The shaded blue regions indicate time points with significant reach deficits (*p* < 0.05, relative to the pre-lesion baseline values), while the white regions represent sessions with no reach deficits. Lines with arrows indicate days where performance was not significantly different from baseline. The dashed lines indicate the mean pre-lesion baseline values. (E) Illustration of the object lifting task behavior setup. Animals were trained to lift and hold an object to test their grasping performance. (F) Grasp durations for monkey N (top) and monkey Hr (bottom) during pre- and post-lesion sessions. The post-lesion sessions are from days with non-significant reaching deficits (see D and E).

We demonstrate the effects of M1 lesions on the reaching performance of two macaques, identified as monkey N and monkey Hr, using a touchscreen-based reaching task (Figure 1A). Both animals were initially trained to proficiency, where they demonstrated consistent reaching behavior (Figure 1B, left). After a targeted M1 lesion (STAR Methods; Figure 1C), their performance was assessed to evaluate the extent and nature of the resulting reach deficits as well as their subsequent recovery. In the immediate period following the M1 lesion (i.e., early post-lesion, ~days 0–14), both monkeys exhibited significant deficits in their reaching ability (Figure 1B, middle). The reach duration and path lengths during reaching attempts increased shortly after the lesion, suggesting that while the animals could still complete the task, their movements were no longer as direct or controlled. This is evident by the reach durations and path lengths in both monkeys (N and Hr) in later sessions (Figure 1D). However, despite the initial impairment, both animals showed recovery over time.

One possibility is that there are changes in kinematic variance. We performed a Levene’s test to test for differences in variance and observed that the variance post-lesion did not generally differ from pre-lesion sessions. In one monkey (N), we observed no significant difference in the variance in trial duration (post-lesion, day 10 *p* = 0.0013, day 11 *p* = 0.15, day 14 *p* = 0.06, day 15 *p* = 0.75, and day 17 *p* = 0.13) and path length (post-lesion, day 10 *p* = 0.08, day 11 *p* = 0.08, day 14 *p* = 0.06, day 15 *p* = 0.46, and day 17 *p* = 0.08) values during the “recovery” sessions. In the second monkey (Hr), we found that the variance in path length slightly increased for two sessions after recovery but then was at baseline in the later sessions of the recovery period (post-lesion: day 8 *p* = 0.06, day 11 *p* = 0.64, day 13 *p* = 0.14, day 14 *p* = 8.14e−06, day 16 *p* = 3.23e−09, day 20 *p* = 0.86, and day 21 *p* = 0.13). The variance in reach durations, on the other hand, remained similar to pre-lesion (post-lesion, day 8 *p* = 0.32, day 11 *p* = 0.15, day 13 *p* = 0.15, day 14 *p* = 0.93, day 16 *p* = 0.05, day 20 *p* = 0.01, and day 21 *p* = 0.08) in the recovery sessions.

Thus, despite potential alterations in gross trajectory shape following M1 lesion, trial-to-trial variability in reach duration and path length were largely preserved. With the exception of a transient increase in spatial variance in one animal, variance returned to baseline levels during recovery. These findings suggest that M1 is not required for maintaining the stability of well-practiced gross motor trajectories, consistent with a shift of control to subcortical circuits after extended training.

Overall, the data show that while reaching movements were impaired early after M1 lesions in macaques, reaching deficits were transient. This indicates that, similar to published findings in rats,^7^ skilled reaching actions can recover after a lesion to M1.

### Longer term grasping deficits in an isolated fine motor task

In the same animals, using a separate task that isolated grasping actions (Figure 1E), we further tested for grasping impairments after an M1 lesion. In this task, animals were required to lift an object for a set time to receive a juice reward. A post-lesion grasp duration was significantly impaired compared to the pre-lesion baseline (monkey N, t = −6.6, *p* = 1.04e−09; Hr, t = −3.3, *p* = 7.9e−04, *t* test) at the time when reaching had recovered (monkey N, day 15; monkey H, day 11). This indicates a more persistent deficit in the animals’ ability to perform fine motor tasks (Figure 1F). Together, these findings suggest that grasping, which requires fine motor control, is more vulnerable to disruption. While the motor system can partially adapt to the loss of M1 in tasks involving gross motor movements, the more precise control required for grasping remained significantly compromised.

### Comparison of reaching and grasping deficits in an RGT

**Figure 2.**
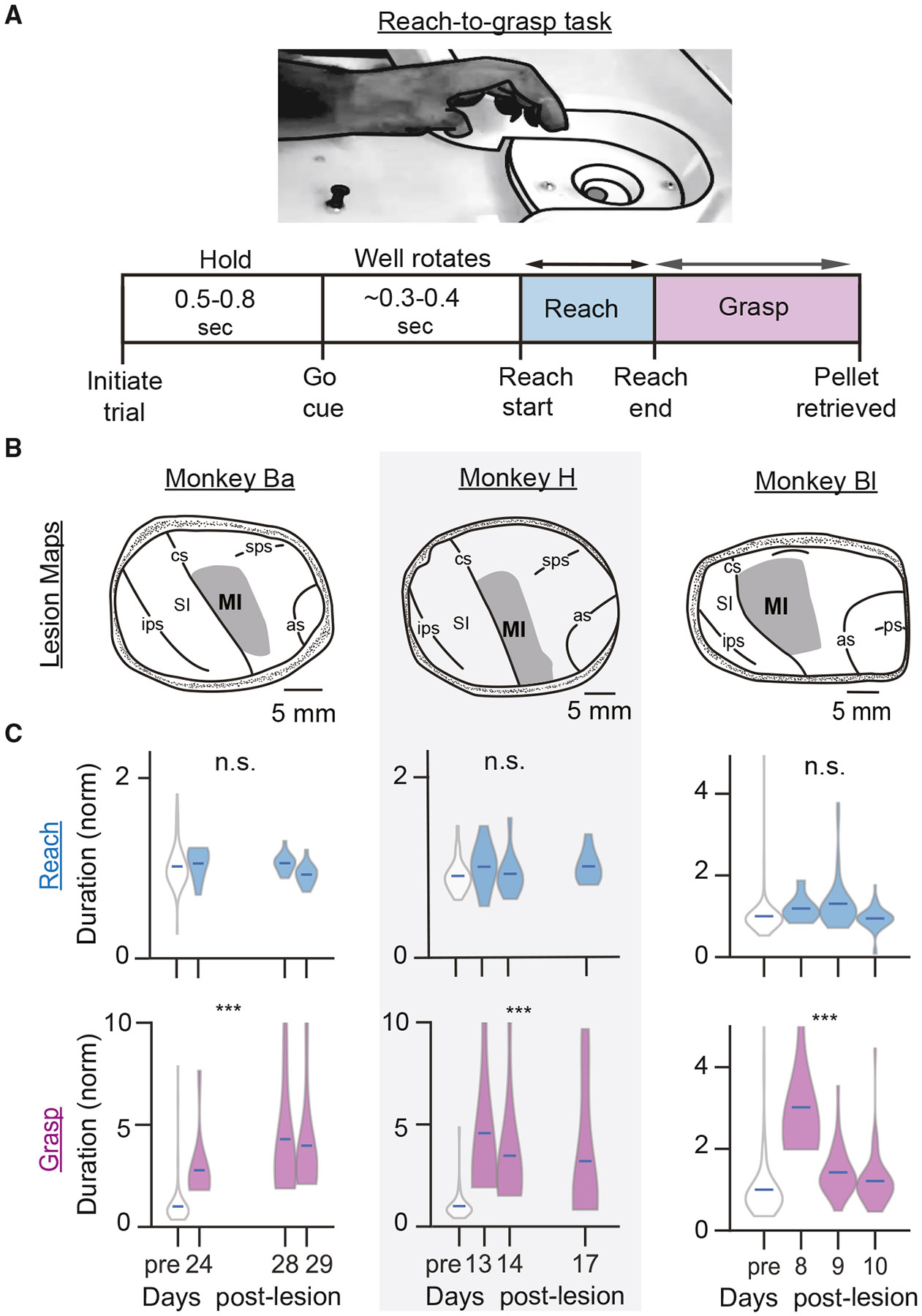
M1 lesion results in long-term grasp deficits during the pellet task in NHPs Animals were trained to retrieve a pellet from a well. Animals were given 7 days to recover post-lesion prior to being tested on the reach-to-grasp task. The first three sessions in which animals could successfully perform at least 5 pellet retrievals were included. Behavior was manually segmented into separate “reach” and “grasp” phases based on when animals initiated movement (reach start), ended the transport of their hand (reach end), and successfully retrieved the pellet from the well (grasp finish). (A) Experimental design. (B) Lesion maps for monkey Ba (top), monkey H (middle), and monkey Bl (bottom). ips, intraparietal sulcus; ps, principal sulcus. (C) Duration of reaching (top) and grasping (bottom) before and after the lesion for monkeys Ba (left), H (middle), and Bl (right). In all three animals, grasping but not reaching deficits are present in post-stroke task performance.

Next, we examined the effects of M1 lesions on a single pellet retrieval task (Figure 2A), which combines reaching and grasping, in a different group of monkeys (*n* = 3, Ba, H, and Bl) (Figure 2B). Their behavior was segmented into three phases: the reaching phase (where the animals transported their hand from the starting position toward the target), the grasp start phase (time of well contact), and the grasp end phase (retraction of the hand to retrieve the pellet from the well). We again observed a difference in the rates of recovery of gross and fine motor control. While reaching performance appeared to be largely unaffected in the long term, as indicated by the lack of significant changes in reach duration after lesion (monkeys Ba, t = −1.8338, *p* = 0.07; H, t = −1.8, *p* = 0.06; Bl, t = −1.7, *p* = 0.1, *t* test), grasping performance was still notably impaired (Figure 2C). This was consistent across all three animals, where significant increases in grasp duration (monkeys Ba, t = −4.0, *p* = 7.4e−05; H, t = −6.7, *p* = 6.0e−10; Bl, t = −9.3, *p* = 1.22e−16, *t* test) were observed following the lesion. This again indicates that gross reaching movements can recover relatively quickly, while prehension deficits are more prolonged. While our primary focus in this study was to examine the acute effects of lesions (to assess for cortical disengagement), we also found that all animals eventually recovered the grasping function with extensive rehabilitation over a period of weeks and months (data not shown).

### Comparison of reaching and grasping deficits in mice

**Figure 3.**
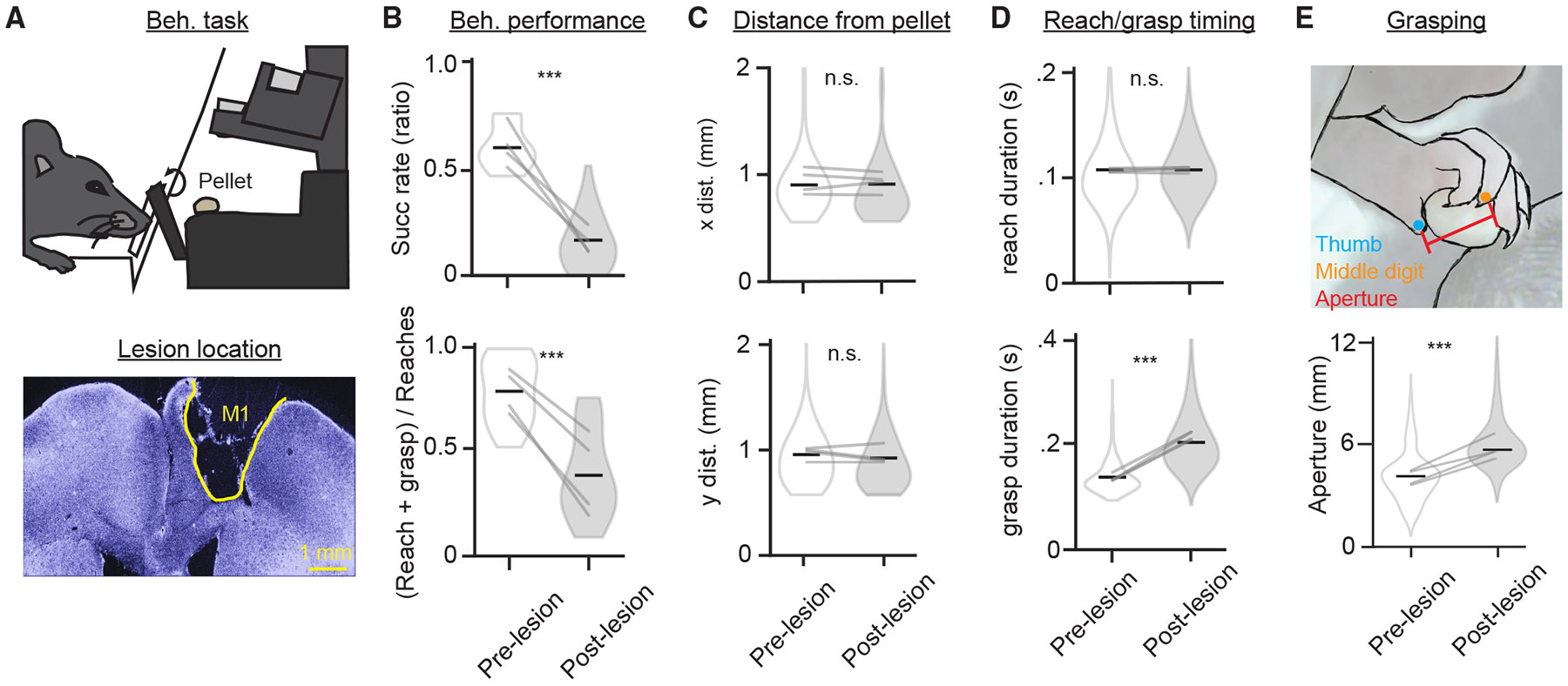
M1 lesion in mouse differentially affects reaching and grasping components Mice were trained to retrieve a pellet from a platform. Behavior was manually segmented into separate “reach” and “grasp” phases based on when animals initiated movement (reach onset), contacted the pellet, and initiated paw retraction (grasp finish). (A) Automated behavior setup (top) and histology showing the site of the lesion (bottom). Scale bars, 1 mm. (B) Behavior performance (pre- and post-lesion) in a pellet reach/grasp task showing the success rate for all mice (*n* = 4) (top) and the fraction of reaches where the mice also initiated a grasp (bottom). (C) Violin plots showing the endpoint distance of the paw in the *x* axis (top) and *y* axis (bottom) from the pellet. (D) Durations of reaches (top) and grasps (bottom) for all mice (*n* = 4). (E) Kinematic measure of grasping showing how the aperture was measured (top), and the pre- versus post-lesion aperture values (bottom).

To match the training duration that is reported to result in disengagement of M1,^7,8^ mice were trained daily for 8 weeks on a single pellet retrieval task until they reached plateau performance before inducing an M1 lesion. We used an automated behavior setup (Figure 3A, top) with high-resolution cameras to monitor forepaw kinematics. We then evaluated the long-term effects of M1 lesions (Figure 3A, bottom) on performance in a task that is analogous to that in the macaques (Figure 2). Like the experiments in macaques, the mice’s reaching behavior was segmented into distinct phases: reach (transport of paw toward pellet), pellet contact, and grasp finish (retraction of paw to retrieve pellet).

Following an M1 lesion, the mice exhibited significant impairments in their ability to successfully perform the reach/grasp task; we tracked changes in behavior over a 1-month period. The success rate—defined as the ratio of successful pellet retrievals to total attempts—was markedly reduced after lesion compared to pre-lesion levels (success rate: pre-lesion = 0.6 ± 0.09, post-lesion = 0.16 ± 0.13, *p* = 7.4e−32, z = −11.846, linear mixed effects model [LMM]) (Figure 3B). This decline in success rate highlights the impact of M1 lesions on the overall motor performance of the mice, particularly in tasks requiring fine motor skills. While the success rate is a coarse measure that can be affected by other factors—such as inaccurate targeting of the pellet—this was not the case as the mice consistently targeted the pellet after M1 lesion similarly to pre-lesion (*y* axis diff., pre-lesion 0.8 ± 0.27 mm, post-lesion 0.79 ± 0.2 mm, *p* = 0.8, z = −0.303; *x* axis diff., pre-lesion = 0.95 ± 0.3 mm, post-lesion 0.9 ± 0.25 mm, *p* = 0.33, z = −0.965, LMM) trials (Figure 3C). We did, however, observe that mice failed to initiate a grasp after contacting the pellet more often in post-lesion trials than in pre-lesion (no grasp: pre-lesion = 0.78 ± 0.14, post-lesion = 0.39 ± 0.2, *p* = 5.9e−14, z = −7.5, LMM) trials (Figure 3B, bottom).

To further explore the precise cause of increased task failures after the lesion, we examined several kinematic measures to determine how consistently the mice performed the reach/grasp task following the lesion. Analysis of reach and grasp durations revealed that, like the findings in macaques, the grasping phase was more severely affected than the reaching phase. The grasp durations after lesion were significantly longer than those observed pre-lesion (grasp duration: pre-lesion = 0.03 ± 0.013, post-lesion = 0.07 ± 0.03, *p* = 3.6e−74, z = 28.1, LMM) (Figure 3D, bottom), indicating a specific disruption in the fine motor control required for successful pellet retrieval. In contrast, reach durations were not impaired (reach duration: pre-lesion = 0.10 ± 0.03, post-lesion = 0.10 ± 0.02, *p* = 0.4, z = −0.896, mixed-effect model) (Figure 3D, top), suggesting a differential impact of M1 lesions on the two components of the motor task.

Additionally, to explore the nature of the grasping deficits, a kinematic analysis focused on the digit aperture of the forepaw during the pellet grasp (Figure 3E, top) was performed. The analysis revealed that the grasp aperture was significantly altered after the lesion (aperture: pre-lesion 3.8 ± 1.3 mm, post-lesion = 5.3 ± 1.3 mm, *p* = 7.9e−69, z = 17.534, LMM), with wider, more variable apertures after the lesion. It should be noted that while the paw aperture was impacted after the lesion, the lesion did not cause a complete loss of function, as the mice sometimes performed successful grasps, as shown by a non-zero success rate and appropriate (i.e., consistent with the pellet diameter) aperture values. Collectively, our results show that an M1 lesion impacted the mice’s ability to precisely and consistently perform the grasping component of the task.

### M1 lesion disrupts transition probabilities in mice and macaques

**Figure 4.**
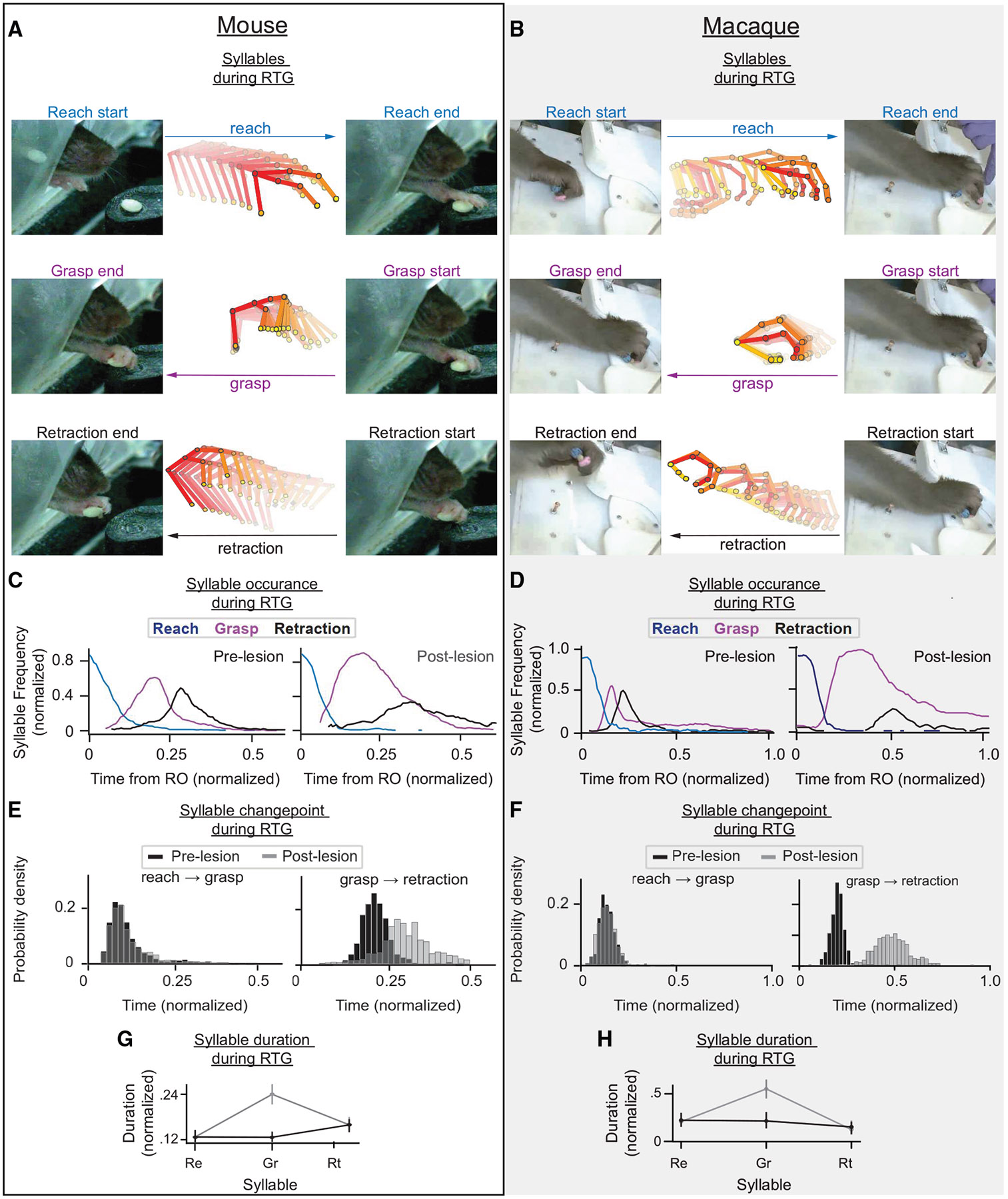
Conserved role of M1 in facilitating transition between reach and grasp (A) Example images of the syllables identified by MoSeq for the three phases of the mouse reach/grasp task movements (reach, grasp, and retraction). (B) Example images of the syllables identified by MoSeq for the three phases of the monkey reach/grasp task movements (reach, grasp, and retraction). (C) Distribution of normalized syllable counts through time for each of the three syllables during mouse pre- and post-lesion trials. Post-lesion is ~2 weeks post-lesion. (D) Distribution of normalized syllable counts through time for each of the three syllables during monkey pre- and post-lesion trials. Post-lesion data are collected after recovery of the reaching phase. (E) Change points indicate the normalized frame at which reaches transition to grasp or grasp transitions to retraction in mice, showing stable reach→grasp timing after lesion and a rightward shift in grasp→retraction reflecting prolonged dwell in the grasp state post-lesion. (F) Change points indicate the normalized frame at which reaches transition to grasp or grasp transitions to retraction in monkeys, showing stable reach→grasp timing after lesion and a rightward shift in grasp→retraction reflecting prolonged dwell in the grasp state post-lesion. (G) Normalized durations of each syllable for pre-lesion (black) and post-lesion (gray) mice trials. (H) Normalized durations of each syllable for pre-lesion (black) and post-lesion (gray) monkey trials.

To understand how M1 lesions disrupted the reach/grasp task sequence, we explored the effects that the lesions had on the timing and occurrence of the task sub-movements^28^ in both mice and macaques. This allowed us to perform a comparative analysis of how M1 damage affects these movements across species. In both species, the task was segmented into key phases: reach, gasp, and retraction (Figure 4A). To quantify these disruptions after an M1 lesion, we employed an unsupervised machine learning method, Keypoint-MoSeq (motion sequencing),^29^ to parse the animals’ behavior into discrete sub-second motifs, referred to as syllables.

Using Keypoint-MoSeq, we reliably identified three movement phases—reach (Re), grasp (Gr), and retraction (Rt)—during the performance of the pellet-reaching task (Figure 4A). In both macaques and mice, pre-lesion trials showed consistent syllable occurrence over time (Figure 4B, top), reflecting stable and proficient task execution. Following the M1 lesion, however, we observed clear disruptions in the temporal structure of the reach-to-grasp sequence in both species. Specifically, the animals’ ability to reliably grasp the pellet was impaired, as evidenced by a broader temporal distribution of the grasp syllable occurrence (Figure 4B, bottom), indicating increased grasp dwell time. In contrast, the reach phase was largely unaffected by the lesion, with post-lesion reach occurrence distributions remaining similar to pre-lesion trials.

To quantify changes in movement sequencing, we performed changepoint analysis to identify the frame at which animals transitioned between sub-movements. We found no significant changes in the reach-to-grasp transition following lesion in mice (pre-lesion = 0.15 ± 0.06, post-lesion = 0.14 ± 0.07, *p* = 0.33, z = −1, LMM; Figure 4E, left) and macaques (pre-lesion = 0.24 ± 0.045, post-lesion = 0.23 ± 0.09, *p* = 0.08, d = 0.07; Figure 4F, left), indicating that the relative timing of this transition was largely preserved. In contrast, the grasp-to-retraction transition was significantly delayed after lesion in mice (pre-lesion = 0.12 ± 0.05, post-lesion = 0.23 ± 0.1, *p* < 0.001, z = −41, LMM; Figure 4E, right) and macaques (pre-lesion = 0.21 ± 0.06, post-lesion = 0.48 ± 0.09, *p* < 0.001, d = 0.9, KS test; Figure 4F, right), indicating that this transition occurred later relative to pre-lesion trials.

We next examined the duration of each sub-movement (syllable). The average normalized duration of the grasp phase was significantly longer post-lesion compared to pre-lesion in both mice (pre-lesion = 0.13 ± 0.05, post-lesion = 0.24 ± 0.09, *p* < 0.001, z = −17, LMM) and macaques (pre-lesion = 0.23 ± 0.1, post-lesion = 0.51 ± 0.1, *p* < 0.001, z = 7.2, LMM). In contrast, the durations of the reach and retraction phases remained unchanged in mice (reach, pre-lesion = 0.12 ± 0.07, post-lesion = 0.13 ± 0.08, *p* = 0.20, z = −1.2, LMM; retraction, pre-lesion = 0.14 ± 0.06, post-lesion = 0.14 ± 0.07, *p* = 0.4, z = −0.80, LMM) (Figure 4G) and macaques (reach, pre-lesion = 0.25 ± 0.1, post-lesion = 0.24 ± 0.08, *p* = 0.13, d = 0.18, KS test; retraction, pre-lesion = 0.19 ± 0.1, post-lesion = 0.18 ± 0.1, *p* = 0.55, d = 0.12, KS test) (Figure 4H).

Together, these results demonstrate that M1 lesions selectively disrupt grasping behavior in both mice and macaques, leading to prolonged grasp duration and delayed transitions to retraction. This cross-species effect highlights the critical role of M1 in coordinating smooth transitions between grasp and retraction phases during skilled motor behavior.

### Long-term engagement of mouse M1 projection neurons

**Figure 5.**
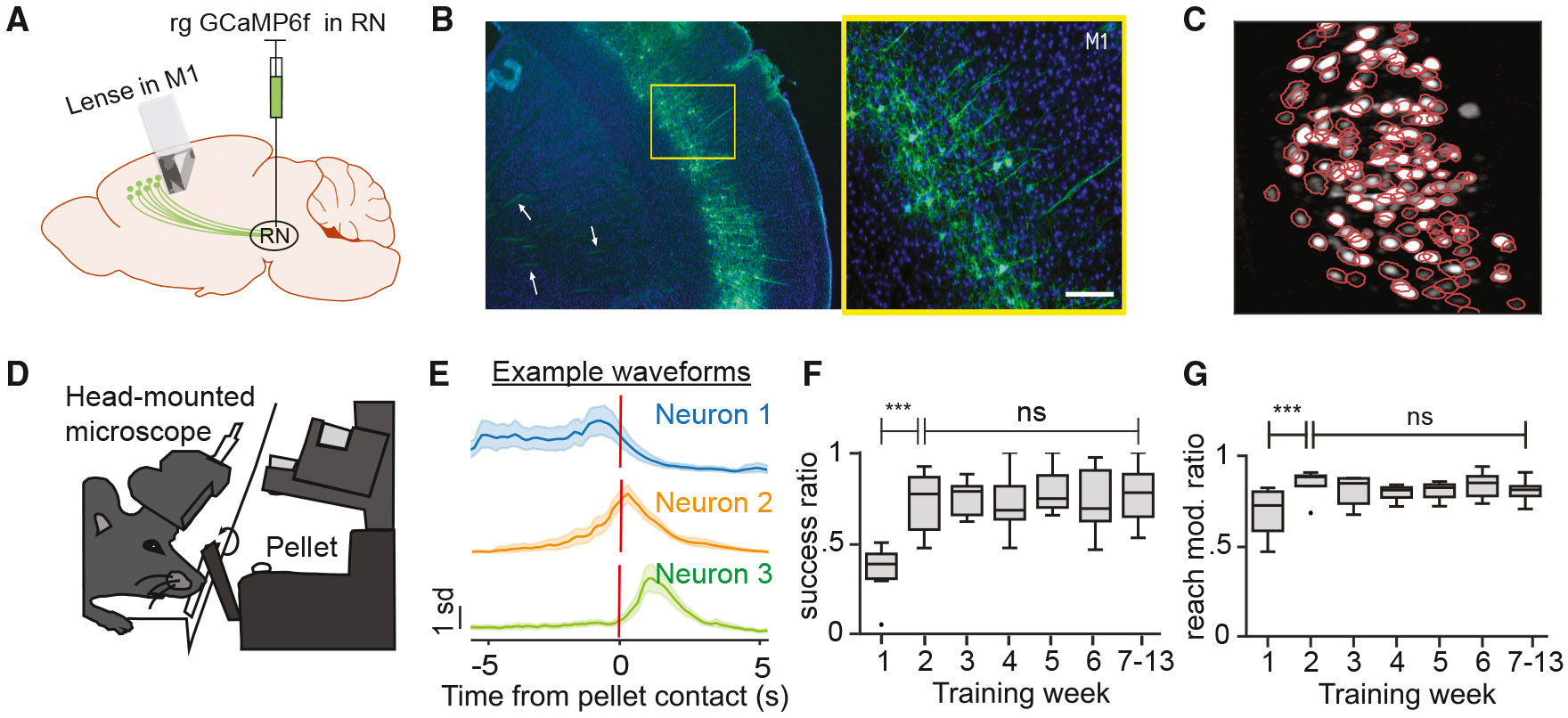
Long-term recording of mouse M1 projection neurons during reach to grasp task (A) Illustration showing injection of retrograde GCaMP6f in red nucleus (RN) and GRIN lens implantation in M1. (B) Histology showing GFP+ layer 5 corticorubral projection neurons in M1. Scale bars, 100 μm. (C) Example FOV recorded using our calcium imaging miniscope detects active projection neurons. Red circles highlight neurons detected by CNMFE (CaImAn). (D) Illustration of reach/grasp task in freely behaving mice using head-mounted miniscope. (E) Example single-session trial-averaged ΔF/F traces from three individual M1-RN projection neurons from one single reach-to-grasp session (shaded, SEM; red line, time of pellet contact). (F) Success ratio for all mice (*n* = 4) during extended task training. (G) Fraction of task-modulated neurons for all mice during training.

Past work also indicated that a reduction in task-related neural activation is consistent with disengagement of M1 in movement control; this analysis was conducted only in upper cortical layers 2/3. However, we specifically hypothesized that examining the projection-specific output of M1, i.e., lower cortical layers, might be more reflective of engagement. We thus analyzed the activity of M1 layer 5 projection neurons during long-term reach-to-grasp learning/skilled performance in intact mice. We thus performed retrograde injection of the calcium indicator GCaMP6f into the red nucleus (RN), a key target of M1 that is implicated in reach-to-grasp control^30-32^; we also implanted a GRIN lens in M1 (Figure 5A). This setup enabled the monitoring of M1 projection neurons (Figure 5B), using a head-mounted miniscope in freely behaving mice while they performed the reach/grasp task. The field of view (FOV) captured the activity of these projection neurons during the task (Figure 5C), allowing for quantification of their engagement over time.

The neural activity of the M1 projection neurons was monitored across 3 months of task training (Figure 5D), with particular focus on the period after the mice had reached a performance plateau. Single-neuron trial-averaged responses revealed that these neurons exhibited consistent activity patterns during the task, even after extensive training (Figure 5E). The success ratio for all mice during the learning of the pellet task was examined, showing an improvement in task performance over the first 2 weeks of training (Figure 5F). As the mice became proficient, the fraction of task-modulated neurons (i.e., neurons whose activity was significantly correlated with the task) increased (Figure 5G). This suggests that M1 projection neurons became more specifically recruited to the task as training progressed. After performance plateaued, this fraction remained stable over the subsequent 13 weeks of monitoring, indicating sustained engagement of M1 layer 5 projections to the RN during long-term skilled performance.

### Stable activity patterns in tracked M1 projection neurons

**Figure 6.**
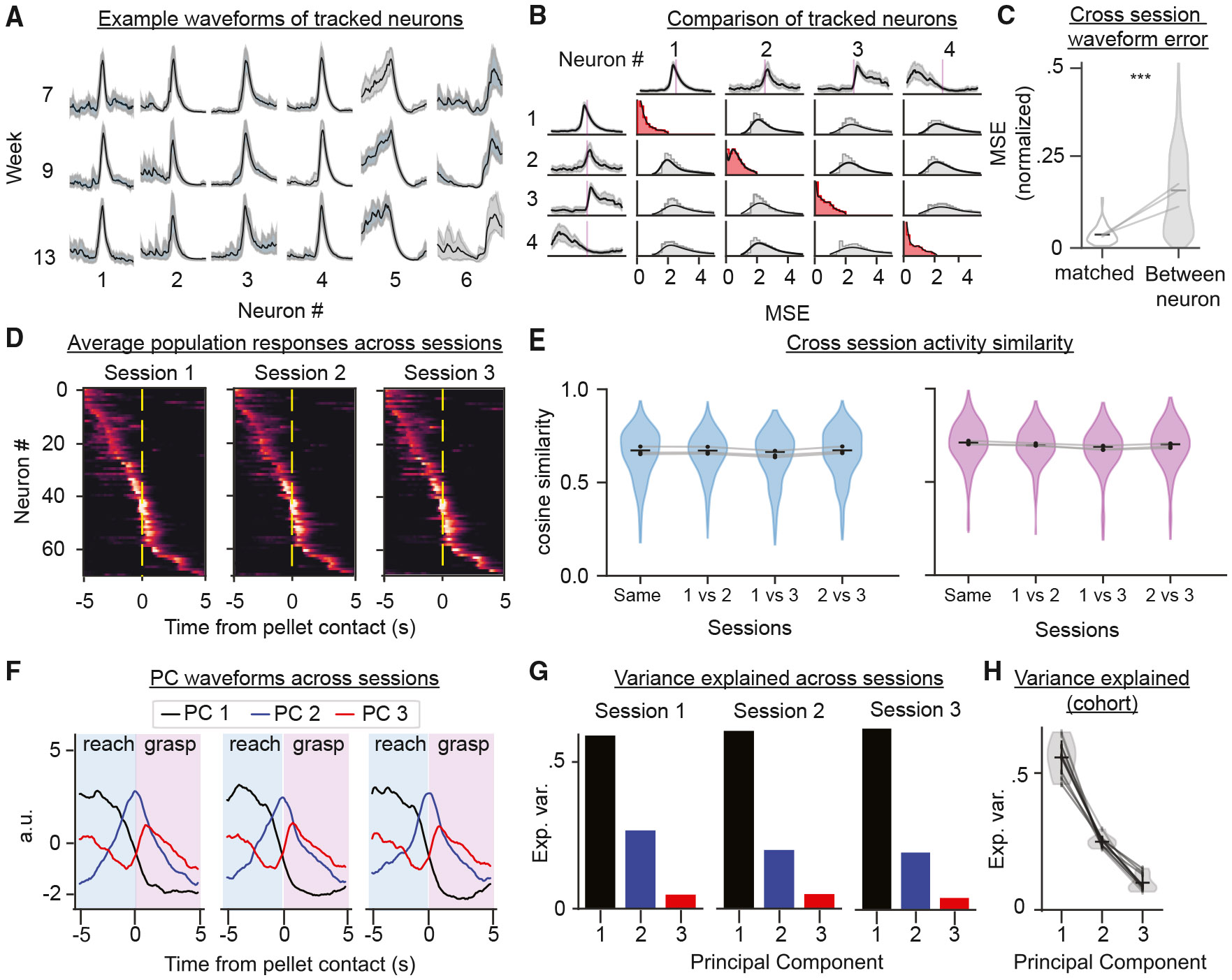
Stability of individual projection neurons and population dynamics (A and B) (A) Six example tracked neuron peri-event waveforms and (B) distribution of pairwise mean-squared-error (MSE) values (individual plots) for example neurons. The diagonals show the distributions of MSE values for the same neuron across days. (C) Distribution of MSE for the traces of every neuron compared to itself across sessions (left) and the MSE between different neurons (right). (D) Example activity profiles for tracked neurons matched across sessions during late learning. (E) Similar calcium activity responses across trials (and sessions) for both the reach and grasp components of the task. (F) Time course of PC activation for the same three sessions from (C). (G) Explained variance for those same sessions in (F). (H) Variance explained by the top 3 principal components (PCs) for the respective sessions (*n* = 3). Violin plots show the distribution of explained variance for each PC, and each line shows the variance for each session.

It is possible that while the overall M1-RN projection neuron population activity remains engaged to the task, specific neurons exhibit variability, session to session. We thus tracked individual neurons across sessions (spanning more than a month) during late learning (after the mice reached a performance plateau). Tracked neurons exhibited remarkably stable activity patterns across sessions, particularly across later sessions (weeks 7–13). The example peri-event waveforms of tracked neurons highlighted this consistency, showing responses and similar waveforms across sessions during the late training phase (Figure 6A).

To ensure that we effectively tracked the same neuron across days, we measured the mean squared error (MSE) between and across neurons (Figure 6B). To assess trial-to-trial waveform similarity of each tracked neuron, we compared the observed trial-by-trial MSE across sessions to a null distribution generated by time-shifting trials. The observed MSE values (MSE: same neuron across sessions = 0.02 ± 0.05) were significantly lower than chance (MSE: null = 0.1180 ± 0.0768, z = −11.73, *p* < 0.001, LMM), indicating that individual neurons exhibit reliable and reproducible activity patterns across trials. Additionally, when comparing the MSE of tracked neurons across sessions to the MSE when compared to other neurons, we would anticipate lower MSE between the same neurons’ waveforms across sessions and higher MSE when comparing the waveforms of different neurons. Indeed, we found that the MSE between the waveforms of tracked neurons across sessions was low for all tracked neurons in all mice. Conversely, when comparing the activity patterns between different neurons, there was, on average, a higher MSE (MSE: between neurons = 0.24 ± 0.30, z = 18.217, *p* < 0.001, LMM; Figure 6C). These results indicate that we were able to reliably track neurons across sessions.

To further explore the stability of reach-to-grasp neural activity, we looked at the consistency of the population activity of all tracked neurons. The heatmaps of trial-averaged neural responses (sorted based on the neuron index from the first session) showed robust within-session population structure across weeks of training (Figure 6D). To quantify the consistency of the neural activity during the reach-to-grasp execution, we calculated the cosine similarity of trial-by-trial peri-event waveforms (during reaching and grasping separately) of each neuron within trials and across trials. Mixed-effects models revealed that similarity was significantly above chance for both reach (similarity: 0.607 ± 0.024, *p* < 0.0001, z = 25.134, LMM; Figure 6E, left) and grasp (similarity: 0.687 ± 0.023, z = 29.601, *p* < 0.0001, LMM; Figure 6E, right) when comparing the neural responses across trials within a session. This indicates that neural population activity is highly consistent across trials within the same session for both sub movements. Population activity was also significantly similar to that expected by chance across sessions—reach (similarity: 0.621 ± 0.027, z = 23.193, *p* < 0.0001) and grasp (similarity: 0.701 ± 0.11, z = 3.193, *p* < 0.001)—indicating strong stability of reach and grasp representations across sessions, spanning a month well after the mice mastered the task. These findings provide evidence for the long-term stability of projection neurons that are continuously engaged during the reach-to-grasp execution in mice.

In addition to the single neuron analysis above, we also performed population-level analysis to determine if the population “co-firing” the activity patterns remained consistent across sessions. Specifically, we quantified the covariation structure among simultaneously recorded neurons using principal component analysis (PCA) of the tracked neurons, which provided insights into the overall single-trial population dynamics of M1 neurons during the task.^33,34^ PCA allowed us to reduce the high-dimensional neural activity into a small number of dominant components that capture the majority of shared variance across the population. When examining the variance explained by the top three principal components, we observed that the variance explained for each component remained consistent across sessions (Figures 6F and 6G) during late learning. Importantly, the relative contribution of each principal component did not systematically shift across days, suggesting that the dominant modes of coordinated population activity were preserved (Figure 6H; *n* = 3 animals). Together, these findings suggest that the population dynamics of projection neurons underlying task performance were stable across sessions in the late learning phase, consistent with the presence of a consolidated and reproducible neural population code.

## DISCUSSION

This study tested whether apparent species differences in M1 function, particularly with respect to gross and fine motor control, reflect true biological divergence or could partially be due to differences in task demands. Across macaques and mice, M1 lesions produced similar patterns: gross reaching recovered relatively quickly, whereas fine grasping deficits persisted. Detailed kinematic and sequencing analyses revealed conserved disruptions in the reach-to-grasp transition in both species. Long-term recordings further showed that layer 5 M1 projection neurons remain stably engaged during skilled performance, arguing against true cortical disengagement. These findings highlight fine motor control as a key, conserved role of M1 across species. Overall, the work emphasizes that task design is essential for cross-species comparisons and supports the translational relevance of mouse models for motor recovery.

### Anatomical versus functional differences between mice and macaques

Despite the similarity in the timescales of recovery of gross and fine motor control, there are clear anatomical differences between rodents and NHPs.^1,35^ Moreover, unlike the rodent M1, the macaque M1 has been found to have at least two sub-regions; the “new” M1 has been defined to have projections to the ventral segments of the spinal cord with direct synapses onto motoneurons.^36^ It has also been implicated in controlling fine movements. In contrast, the “old” M1, the rostral part of the precentral gyrus, is implicated in more general limb and wrist movements.^37,38^ Furthermore, there are different profiles of descending connections between the two species.^1^ For example, a recent study compared the corticospinal projectome in rats and macaques.^1^ In rats, corticospinal projections were broadly distributed to sensory and integrative regions, including the striatum, brainstem, and both upper and lower spinal cord. In contrast, macaque corticospinal projections had a dominant output to lower cervical spinal cord regions and a higher prevalence of direct cortico-motoneuronal connections, highlighting their specialization in fine motor control. Transsynaptic analyses showed that while rats target diverse spinal interneurons, macaques predominantly target alpha motor neurons and inhibitory interneurons, likely reflecting evolutionary shifts in motor control specialization. Importantly, unlike in the rodent, the macaque motor network is also characterized to have multiple cortical areas, e.g., supplementary motor area and dorsal and ventral premotor areas that project to the spinal cord and appear to play an important role in reach-to-grasp actions.^4,39-42^

How do we then reconcile these anatomical and functional differences? One possibility is that M1 has a generally conserved role in regulating the spatial and temporal activation of descending pyramidal projections to the brainstem and spinal cord, while also coordinating descending extrapyramidal pathways. However, there are differences in how this information is delivered. For example, in rodents, M1 may broadcast to a wider array of subcortical and spinal targets, reflecting a more distributed control strategy. In contrast, primates appear to utilize both a parallel and a hierarchically organized system, where partially specialized motor areas exert targeted control over reach to grasp control^37,43^; this can, for example, allow complex control that integrates the complex visuomotor processing in the grasp network.^43-45^

### Timescales of recovery

The differences in the timescales of recovery for gross versus fine motor control following M1 lesions in both rodents and macaques likely reflect fundamental distinctions in the underlying neural mechanisms and circuit-level demands. Gross motor functions, which involve larger, less precise muscle groups such as those used in reaching or transporting the limb, appear to recover rapidly even in the absence of targeted rehabilitation.^7^ This suggests that such actions can be mediated by a distributed motor network that has the potential to compensate for the loss of M1.^4,20^ In both species, existing cortical regions (e.g., residual motor and premotor areas) and subcortical structures, including the basal ganglia^12,15,46^ and reticular formation in the brain-stem,^47^ may relatively rapidly compensate for M1 loss by leveraging preserved connectivity. While the exact mechanisms remain unclear, homeostatic plasticity is one possibility.^11^

In contrast, fine motor control, such as grasping and digit individuation, likely requires highly specific, temporally precise output patterns,^48^ often involving direct cortico-motoneuronal connections, particularly in primates.^36^ The recovery of these skilled movements is slower and appears to depend on mechanisms of neural plasticity, including the recruitment and reorganization of residual cortical tissue, functional remapping of peri-lesional regions, and the strengthening of alternative ticospinal or cortico-subcortical pathways.^49^ In the context of extensive M1 damage, fine motor control may also rely on the compensatory activation of secondary motor areas, such as the supplementary motor area and possibly even contra-lesional homologous regions.^20,22^ Notably, analogous electrical stimulation of premotor areas in both rats^50-52^ and macaques^25,27,53^ has shown improvements in reach-to-grasp function after an M1 lesion, supporting the importance of cortical input in compensating for lost primary motor output and coordinating skilled forelimb movements.

Furthermore, rehabilitation plays a critical role in facilitating these processes, promoting synaptic strengthening and activity-dependent changes in connectivity within spared networks.^21,54^ Thus, while gross motor functions may exploit preexisting, resilient motor architecture for more rapid functional restoration, fine motor recovery may involve a more prolonged process of circuit-level adaptation and re-engagement, highlighting distinct recovery mechanisms with implications for both basic neuroscience and the design of targeted therapeutic interventions.

### Impact of M1 lesions on motor variability

Another important aspect of our study was to understand the role of M1 in regulating movement variability.^55^ After the lesion, both mice and macaques exhibited increased variability and fragmentation in their motor sequences, particularly during the transition from reaching to grasping. This was supported by the movement patterns captured through MoSeq analysis. The disruption of smooth transition probabilities between syllables after a lesion suggests that M1 contributes to the predictability of motor sequences, a feature that may be important for the reliable execution of skilled movements. The increase in motor variability following M1 lesions may also reflect a loss of the regulatory control that M1 exerts over movement execution. Previous studies have suggested that variability in neural activity can be beneficial during the early stages of learning, allowing for exploration and adaptation.^55,56^ However, our findings indicate that once a motor skill, particularly involving prehension, is well-learned, M1 may shift its role toward minimizing variability to ensure consistent and precise performance.^12,57-61^ The post-lesion increases in variability observed in our study could therefore represent a regression to a less stable, more exploratory state, highlighting the importance of M1 in maintaining motor precision.

### M1 projection neurons and their role in motor control

Our calcium imaging experiments revealed that M1 projection neurons remain highly engaged during the skilled motor task, even after extended training. The stable activity of these neurons was observed throughout 12 weeks of training, even after the animals reached a performance plateau in the RGT. This stability suggests that M1 projection neurons play an ongoing role in motor control, contributing to the precise coordination required for tasks involving fine motor skills, such as grasping. The consistency of M1 projection neuron activity across sessions further supports the idea that these neurons are integral to maintaining task performance, particularly during the fine motor components of the RGT. The stable engagement of M1 projection neurons also contrasts with the idea that M1 becomes disengaged in task execution over time. Consistent with this, the extent of injury to the descending white matter is predictive of recovery of hand function.^62^ Thus, understanding specifically how deeper layer projection neurons change after injury may be an important new direction to understand recovery after stroke.^63,64^

How do we reconcile this with reports wherein superficial layers showed reduced modulation with long-term training^8^? Notably, that study employed a likely open-loop, gross motor lever task, raising the possibility that the apparent reduction in superficial-layer engagement reflects task demands rather than a general decline in M1 involvement. In this context, it remains quite possible that the upper cortical layers follow a different pattern under conditions, requiring greater sensory feedback and fine motor control.^6,36^ Given that fine dexterous behaviors—particularly grasping—depend strongly on rapid peripheral feedback, superficial-layer activity during long-term training may remain more strongly engaged in feedback-dependent tasks.^65^ Thus, differences across studies may also reflect variation in feedback and control demands rather than laminar specificity per se, a possibility that warrants direct testing in future work.

### Limitations of the study

First, our neural measurements rely on calcium imaging, which has substantially slower dynamics than the underlying spiking activity and the rapid kinematics of reach-to-grasp movements. As a result, calcium signals integrate activity across closely spaced behavioral events, limiting our ability to temporally dissociate neural contributions to individual subcomponents of the reach and grasp that occur within hundreds of milliseconds of one another, particularly in rodents.

Second, although framing recovery in terms of gross versus fine motor control is conceptually useful, this distinction may be a simplification. NHPs possess substantially greater hand control and digit individuation than rodents, reflecting more elaborate corticospinal and sensorimotor specializations.^38,43,66^ When extended to humans, these differences become even more pronounced, with markedly higher levels of dexterity and independent finger control.^44,66^ Accordingly, while we observe conserved recovery trends across species, more nuanced fine motor outcomes, such as precise finger individuation,^67,68^ are likely to exhibit meaningful species-specific differences. Moreover, although mice and rats are grouped together,^7-10^ they may also exhibit some species-specific differences in reach kinematics and motor-skill learning strategies.^69,70^

Finally, our behavioral tasks primarily are likely to involve open-loop automatic movements without explicit perturbations or changing task demands.^34,56^ As a result, our findings do not address how M1 lesions affect feedback-dependent gross motor behaviors or adaptation to large changes in task parameters. Notably, one of the past studies on disengagement also lesioned premotor cortex in addition to M1.^7^ It is important to also consider the role of premotor regions in learning and skill consolidation^71,72^ and for compensatory control.^4^ These factors should be considered when generalizing our results to more complex behaviors, e.g., those that require extensive feedback or rehabilitation contexts.

This study provides evidence for the essential role of M1 in skilled control of the hand, particularly in the regulation of movement precision and variability. The findings highlight the challenges associated with recovering fine motor functions following M1 lesions and highlight the importance of M1 in maintaining the stability of learned motor behaviors. The conservation of these mechanisms across species reinforces the relevance of rodent models for studying motor function, recovery, and therapeutic strategies.^4,50,73,74^

## STAR★METHODS

### EXPERIMENTAL MODEL AND STUDY PARTICIPANTS DETAILS

#### Mice

Experiments were approved by the Institutional Animal Care and Use Committee at the San Francisco VA Medical Center. We used eight wildtype C57BL/6J (Jackson Laboratory, Jax #000664, *Mus musculus*) 10–12 weeks old adult male mice for lesion (*n* = 4) and calcium imaging (*n* = 4) experiments. Only male mice were used to minimize behavioral variability. The mice were group housed by cohort, including the recovery period after surgery. Mice were kept under controlled temperature (65°–75°F) and humidity (40–60%) with a 12-h light/dark cycle; lights on at 06:00 a.m.

#### Macaques

Experiments were conducted in compliance with the NIH Guide for the Care and Use of Laboratory Animals and were approved by the University of California, Davis Institutional Animal Care and Use Committee. A total of five male adult healthy rhesus macaques (*macaca mulatta)* were used in experiments. Animals were 5–6 years old and 12.0–15.0 kilograms. Four animals were pair-housed and one animal was singly housed.

### METHOD DETAILS

#### M1 Photothrombotic lesion in mice

Focal stroke of the entire primary motor cortex (M1) contralateral to the dominant forepaw was performed using LED illumination of 20 mg/kg Rose Bengal dye under isoflurane (1–2%) and body temperature maintained at 37C with a heating pad. A 2.5 mm × 1.6 mm LED (Digi-Key Electronics) was placed over the center of the M1 region (1.6 mm lateral and 0.5 mm anterior to bregma). Five minutes after Rose Bengal (20 mg/kg) i.p. injection, LED was illuminated (0.125 mA) for 20 min, resulting in formation of a permanent stroke lesion in primary motor cortex.

#### Lesion in macaques

Following task training, animals underwent a stroke-induction and microelectrode array implantation surgery. Preoperatively, animals were sedated with ketamine hydrocholoride (10 mg/kg), administered atropine sulfate (0.05 mg/kg), prepared and intubated. They were then placed on a mechanical ventilator and maintained on isoflurane inhalation (1.3–1.6%). Animals were positioned in a stereotactic frame (David Kopf Instruments, Tujunga, CA) and administered mannitol (1.6 g/kg) intravenously prior to the craniotomy (note that two animals did not receive mannitol in an effort to improve microelectrode array insertion). A skin incision, bone flap, and dural flap were made over the lateral frontoparietal convexity of the hemisphere and the caudal region of the frontal lobe and rostral region of the parietal lobe was exposed unilaterally. After cortical exposure, the lesion was induced using surface vessel coagulation/occlusion followed by subpial aspiration. Specifically, the lesion extended dorsally to a horizontal level including the precentral dimple (the lateral-most part of the of the M1 leg area) and ventrally to the central sulcus genu (the dorsal most part of the M1 face area). Following surgery, animals were administered analgesics and antibiotics, and were carefully monitored post-operatively for 7 days.

#### Macaque pellet retrieval task

A custom-built, automated pellet retrieval task was used to assess forelimb function in three animals. Briefly, animals were seated in a primate chair outfitted with a door allowing them to interact with the pellet task. To initiate a trial, animals touched a “start screw” that was connected to a capacitive touch sensor. Holding the screw for the random hold time (uniformly distributed between 0.5 and 0.8 s) triggered the automated apparatus to release an edible pellet from an automated pellet dispenser (80209–190S,142 Lafayette Instrument, Lafayette, IN) into one of five “wells”. All five wells had depths of 5.9mm and diameters of either 13mm, 19mm, 25mm, 31mm, or 37mm, with the smaller wells making the pellet retrieval more challenging for animals. Animals had 5 s to retrieve the pellet from the well before the apparatus would rotate the well out of view. Animals performed 10–100 trials per day, depending on impairment. Two cameras (CM3-U3–13Y3C-CS, Point Gray, Richmond, BC, Canada) were mounted to a stainless-steel platform providing a side and top view of the animal performing the pellet retrieval task. Camera frames and touch sensor activity were synchronized using the electrophysiology recordings system (Tucker-Davis Technology, Alachua FL). The reach data was then fed into a pose estimate algorithm – Deeplabcut (Mathis, A et al. 2018) – to label the hand and digits during each trial.

#### Mice behavioral training

Mice were food-restricted and habituated to an automated plexiglass behavioral box for 2–3 days before starting behavior training. Following habituation, daily training (5x/week for 12 weeks) began in the automated behavioral box. The box was controlled by an Arduino microcontroller and custom MATLAB scripts requiring minimal user intervention. Body weight was measured daily to ensure the mice retained at least 90% of their baseline weight. Each session consisted of 75 trials, regardless of the number of trials in which the mouse attempted a reach. In our automated systems, these sessions typically took place over ~25 min. Reaching behavior was captured with two cameras (lateral and front) which acquired images at 200 Hz. The reach data was then fed into a pose estimate algorithm – DeepLabCut^75^ – to label the hand and digits during each trial.

#### Virus injection and GRIN lens implantation

Prior to surgery, animals were briefly food-restricted and allowed to perform 10–20 reaching trials to determine forelimb preference; following this assessment, animals were allowed to feed freely until the time of experimentation. Virus injection and lens implant surgery was performed under isoflurane (1.6–2%) while the body temperature was maintained at 37°C with a heating pad. For retrograde GCaMP6f injection into the red nucleus, a burr hole was created (0.65 mm lateral and 3.5 mm posterior to bregma) contralateral to the dominant forepaw and then a 32G needle attached to Hamilton syringe was lowered into the brain tissue at a depth of 3.8 mm. Using an automated injection pump, 500 nL of the virus was injected at a flow rate of 100 nL per min. Once the injection was finished, the needle was kept in place for an additional 5 min to prevent backflow. The needle was then slowly removed from the brain and the burr hole was sealed with bone wax.

After the virus injection, 2 additional burr holes were created for 2 skull screws to allow for secure fixation of the Gradient Refractive Index (GRIN) lens and integrated baseplate (Proview integrated prism lens; Inscopix, Palo Alto, CA). A 1.6 mm × 1.6 mm craniotomy was then performed over the primary motor cortex (1.6mm lateral; 0.5mm anterior). After the craniotomy, a 1 mm incision was made in the anterior/posterior orientation with a microblade to minimize displacement of brain tissue while placing the 1 mm × 1 mm prism lens. After insertion of the lens, the lens/baseplate was fixed to the skull using a combination of Metabond and dental cement. Buprenorphine (0.02 mg/kg) was administered for post-operative analgesia and weight was carefully monitored for five days following surgery.

#### Neural recording and behavioral training

Four weeks following virus and lens implantation surgery, mice were again food-restricted and habituated to an automated plexiglass behavioral box for 2–3 days. Following habituation, daily training (5x/week for 12 weeks) began in the automated behavioral box. The box was controlled by an Arduino microcontroller and custom MATLAB scripts requiring minimal user intervention. Body weight was measured daily to ensure the mice retained at least 90% of their baseline weight. Each session consisted of 75 trials, regardless of the number of trials in which the mouse attempted a reach. In our automated systems, these sessions typically took place over ~25 min.

Reaching behavior was captured with a lateral camera which acquired images at 200 Hz. Flashes of an LED (controlled by Arduino) in the field of view were used to synchronize reaching behavior with the task (door opening, pellet drop, and door close) and neural data. A custom python script was used to manually determine the success and reach onset for each trial. Training outcome was assessed by calculating the reach success (number of trials in which a pellet was retrieved into the behavioral box/number of trials in which reach was attempted) for each behavioral session.

#### Calcium imaging

At the beginning of some training sessions (~2x/week), a small head-mounted microscope was attached to the previously implanted prism lens and baseplate (nVoke imaging systems; Inscopix, Palo Alto, CA). Prior to the first imaging session (Imaging FOV: 600μm × 950 μm), light intensity and gain were adjusted to the minimal settings that could allow for identification of fluorescence transients to minimize photobleaching. After these parameters were established, the same settings (LED power and Gain) were used for the remainder of imaging sessions for that animal. Calcium imaging data was acquired using the miniscope at 10 Hz for ~3 months (2–3 sessions/week), and neural data was synced to behavior *post-hoc* using timing pulses generated by a microcontroller. Images were exported as TIFF stacks and fluorescence signals were extracted using CaImAn^59^ imaging data analysis software. After curation of data exported from CaImAn software, custom python scripts were used to evaluate each putative neuron identified by CaImAn software.

#### Immunohistochemistry

Mice were transcardially perfused with 0.9% saline followed by 4% paraformaldehyde (PFA, in 0.2 M PBS) to fix brain tissue prior to its removal from the skull. Brains were post-fixed in 4% PFA for 24 h then cryopreserved in 20% sucrose with sodium azide (Sigma) at 4 C. Using a cryostat (Leica), 50 μm thick histological sections were cut directly onto microscopic slides. Brain sections were washed 3 × 5 min with 0.01M PBS, followed by 1h of blocking and permeabilization with 5% normal goat serum (NGS, Sigma) and 0.3% Triton X-100, diluted in PBS. Primary antibody against green fluorescent protein (anti-GFP, 1:1000, ab13970, Abcam) was applied, diluted in PBS +0.3% Triton +5% NGS, and incubated at 4 C overnight. After rinsing off the primary antibody (3 × 5 min PBS), secondary AlexaFluor antibody (1:500, Life Technologies) were applied for 1h at room temperature followed by 3 × 5 min washed with PBS. Finally, sections were covered with Vectashield mounting medium and stored at 4C prior to imaging with a Zeiss fluorescence microscope.

### QUANTIFICATION AND STATISTICAL ANALYSIS

All statistical analyses were performed using Python (v3.10) unless otherwise noted. Statistical significance was defined as α = 0.05. Data are presented as mean ± SEM unless otherwise stated. n refers to animals unless otherwise specified.

#### Macaque analysis

A custom python script was used to manually annotate the timepoints of interest (reach onset, initial well/platform contact, and the end of well/platform contact during paw retraction) for each reach attempt. Using these timepoints, we segmented the movement into three phases - reach, grasp, retraction to determine the reach and grasp durations for each trial. The reach duration was calculated as the time from reach onset to the time of paw contact with the well. The grasp duration was calculated as the time the animal’s paw came in contact with the well to the time they grasped the pellet grasp and retracted their paw away from the well. All durations were normalized by dividing the movement durations by the average of the pre-lesion durations for each well size. This allowed us to evaluate any behavioral changes that occurred in post-lesion trials relative to the pre-lesion baseline. To test for significance, we used an independent *t* test (SciPy –python) and a *p* value cutoff of 0.05. We tested for significance in reach and grasp durations for each monkey separately.

#### Mouse behavioral analysis

Movement phases were identified using the same approach used in the analysis of the macaque data. Using the time points of interest, we determined the reach and grasp durations for each trial. The reach duration was calculated as the time from reach onset to the time of pellet contact. The grasp duration was calculated as the time of pellet contact to the time of paw retraction away from the platform (on trials where a grasp – i.e., flexion of digits – was attempted). Training outcome was assessed by calculating the reach success (number of trials in which a pellet was retrieved/number of trials) for each behavioral session. All durations were normalized by dividing the durations by the mean of all duration for each stage (pre/post lesion). To test for significance, we used a mixed effects model (statsmodels – python) and a *p* value cutoff of 0.05. We calculated the aperture as the distance from the thumb to the tip of the middle digit. The distances from the pellet were determined by calculating the pixel distance between the tip of the middle digit and the center of the pellet in the x and y coordinates and subsequently converted to mm. To test for significance for both the aperture and the distances from the pellet, we used a mixed effects model and a *p* value cutoff of 0.05.

#### MoSeq analysis

To extract the syllables for each reach, the DeepLabCut output – labeled points through time – were used as inputs to train a Keypoint-MoSeq^29^ model to identify motifs in the behavioral data. The MoSeq model was trained with the complete dataset (pre + post-lesion) to allow for direct comparison between the syllables pre and post-lesion. The input for each model consisted of ~1000 reaches of ~100 frames each for the mouse model and ~500 reaches of ~100 frames for the macaque model. The model was trained for 10,000 iterations – when the - log likelihood was maximized. The trained model identified five syllables in total; two occurred at very low frequency (<0.5%) and were therefore excluded from further analysis. The remaining three syllables robustly captured the key phases of the reach-to-grasp sequence (reach, grasp, and retraction.

Syllable occurrence over the course of each trial was computed by summing the number of times a given syllable occurred at each time point and dividing by the total number of syllable occurrences at that time point. Syllable duration was calculated as the number of frames between syllable onset frames (as determined using functions from the MoSeq package) divided by the video sampling rate (200 frames/s for mice and 50 frames/s for macaques).To quantify lesion-induced changes in behavioral timing, changepoints were additionally calculated relative to each mouse’s pre-lesion baseline. For each transition, the mean pre-lesion changepoint was computed per mouse and subtracted from all trials, such that pre-lesion values were centered at zero. Post-lesion values therefore reflect within-mouse shifts in relative timing.

#### Neural analysis and Statistics

After curation of data exported from CaImAn, fluorescence signals were z-scored across the entire behavioral session. Task modulation of individual neurons was assessed using an ANOVA comparing pre- and post-reach activity for each neuron recorded during the session. Figures showing averages across animals report the mean ± s.e.m. throughout.Statistical significance for both behavioral success rate and the number of modulated neurons was assessed using linear mixed-effects models implemented in Python. These models account for the non-independence of repeated measurements within animals, allowing for robust aggregation and inference across animals.

For assessment of long term stability of the neural activity, neurons were tracked across sessions using the CaImAn ROI registration function and were verified manually. Neural analyses were performed on the subset of neurons successfully matched across all sessions. Neural activity was parsed into trials by extracting 5 s of data before and after pellet contact. To generate heatmaps, trial activity for each neuron was averaged across trials. Neurons were ordered based on the maximum activity amplitude of their average trace over time in the first session, and this ordering was then applied to subsequent sessions.To assess similarity in neural activity across sessions, we computed the cosine similarity of trial-by-trial population activity vector (the flattened neuron x time matrix) across sessions separately for the reach and grasp periods of the trial. A linear mixed-effects model was used to test for significant differences in the activity across sessions.

The mean squared errors between calcium activity of the same neuron across sessions was determined by calculating the average squared difference between trial peri-event waveforms of one session and a second session, for all session pairs. The mean squared errors between neuronal peri-event waveforms of different neurons was determined by calculating the average squared difference between trial peri-event waveforms of each neuron and a different neuron, for all neurons. Dimensionality reduction was performed using PCA (sklearn python package) to obtain the primary components and the variance explained by each component. To determine if there was a difference between the amount of variance explained by each component (across mice and across session) we used a mixed effects model (statsmodels – python) and a *p* value cutoff of 0.05.

## Figures and Tables

**Table:** KEY RESOURCES TABLE

REAGENT or RESOURCE	SOURCE	IDENTIFIER
Antibodies		
anti-GFP	Abcam	Cat #: ab13970; RRID:AB_300798
Bacterial and virus strains		
rAAV2Retro-hSyn-GCaMP6f	UNC Vector Core	https://www.med.unc.edu/vectorcore/in-stock-aav-vectors/; RRID:SCR_002448
Biological samples		
Adult male mouse brain tissue	This study	N/A
Macaque cortical tissue	This study	N/A
Chemicals, peptides, and recombinant proteins		
Rose Bengal dye	Sigma-Aldrich	Cat #: 198250
Isoflurane anesthetic	Veterinary supplier	N/A
Buprenorphine	Veterinary supplier	N/A
Deposited data		
Data related to figures	This study	https://doi.org/10.48324/dandi.001782/0.260421.2247
Experimental models: Organisms/strains		
Mouse: C57BL/6J	The Jackson Laboratory	JAX: 000664; RRID: IMSR_JAX:000664
Rhesus macaque (*Macaca mulatta*)	California National Primate Research Center	N/A
Software and algorithms		
MATLAB	MathWorks	https://www.mathworks.com; RRID:SCR_001622
Python 3.10	Python Software Foundation	http://www.python.org/; RRID:SCR_008394
CaImAn calcium imaging analysis v1.10	https://github.com/flatironinstitute/CaImAn	RRID:SCR_021533
DeepLabCut	http://www.mackenziemathislab.org/deeplabcut	https://github.com/DeepLabCut/DeepLabCut
Keypoint-MoSeq	https://github.com/dattalab/keypoint-moseq	RRID:SCR_025032
Custom code	This study	https://zenodo.org/records/19615415
Other		
nVoke miniscope imaging system	Inscopix	https://www.inscopix.com/nvoke; RRID:SCR_023028
ProView Integrated Prism GRIN Lens	Inscopix	https://www.inscopix.com/
Automated pellet retrieval apparatus	Custom built	This study
Arduino microcontroller	Arduino	https://www.arduino.cc
LED illumination module	Digi-Key Electronics	https://www.digikey.com/

## Highlights

- Rodent and primate recovery profiles are conserved when task demands are matched
- Reaching recovers rapidly after motor cortex lesions, while grasping deficits persist
- Reliability of reach-to-grasp transitions is disrupted, increasing sequence variability
- M1 projection neurons remain stably engaged during long-term skilled motor performance
